# Intergenerational and organ-specific alterations in mitochondrial DNA copy number following preconception irradiation

**DOI:** 10.1016/j.redox.2026.104054

**Published:** 2026-01-30

**Authors:** Ryosuke Seino, Hisanori Fukunaga

**Affiliations:** a Department of Biomedical Science and Engineering, Faculty of Health Sciences, Hokkaido University, Sapporo, Japan; b Center for Environmental and Health Sciences, Hokkaido University, Sapporo, Japan

**Keywords:** Intergenerational effect, Growth-related traits, Mitochondrial DNA copy number, Preconception exposure, Radiation

## Abstract

Ionizing radiation, a potent inducer of redox stress, perturbs both nuclear and mitochondrial genomes, yet how such stress shapes mitochondrial inheritance across generations remains unclear. In this study, we examined intergenerational and organ-specific mitochondrial responses to parental X-ray irradiation in mice. Eight-week-old male and female C57BL/6N mice were exposed to 2 Gy of single whole-body X-ray irradiation before mating, generating paternal-, maternal-, and dual-irradiated lineages. In the parents, peripheral blood-derived mitochondrial DNA copy number (mtDNAcn) transiently increased one day after exposure, consistent with a rapid mitochondrial response to redox stress. In newborn offspring, mtDNAcn displayed clear organ- and parent-of-origin specificity: brain mtDNAcn decreased in paternal- and dual-irradiation lineages, heart mtDNAcn remained unchanged, and liver mtDNAcn showed the most pronounced depletion across all irradiated lineages. No significant inter-organ correlations in mtDNAcn were observed. All irradiated lineages exhibited increased body weight and increased liver weight at birth, with a significant positive association between these traits. Liver weight was negatively associated with hepatic mtDNAcn. Multiple regression analysis further showed that maternal pre-exposure mtDNAcn and offspring hepatic mtDNAcn independently predicted neonatal liver weight. Taken together, these findings demonstrate that preconception irradiation induces acute mitochondrial responses in parents and is associated with intergenerational, organ-specific mtDNAcn dysregulation that manifests as offspring birth outcomes. Parental irradiation perturbs organ-specific mitochondrial genome regulation and predisposes the next generation to altered growth-related traits.

## Highlights

• Preconception irradiation is associated with acute mtDNA responses in parents.
• Offspring exhibit intergenerational and organ-specific alterations in mtDNAcn.
• No coordinated inter-organ mtDNAcn changes are observed in offspring.
• Neonatal body and liver weight are increased across irradiated lineages.
• Reduced hepatic mtDNAcn is associated with increased neonatal liver weight.

## Graphical abstract

**Figure:**
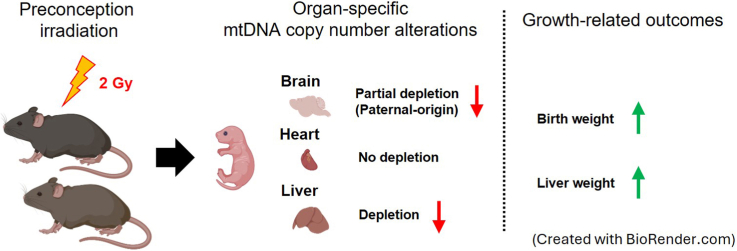

## 1 Introduction

Since the recognition of mitochondrial diseases in 1962 [1], numerous studies have elucidated the physiological and pathological significance of mitochondrial function. Mitochondrial disorders often manifest in organs that are highly dependent on oxidative phosphorylation for energy supply. In the neonatal period, a characteristic “triad” of cerebro-muscular symptoms (e.g., recurrent apnea, convulsions, hypotonia), gastrointestinal and hepatic abnormalities (e.g., vomiting, poor feeding, diarrhea, hepatomegaly), and myocardial dysfunction (e.g., arrhythmias, cardiomyopathy, heart failure) has been described [2]. These organ systems—brain, liver, and heart—are therefore regarded as particularly vulnerable to mitochondrial dysfunction. Accordingly, we focused our analysis on the brain, liver, and heart to determine whether preconception irradiation induces uniform or organ-specific alterations in mtDNA copy number in offspring.

Mitochondrial DNA (mtDNA) is generally more susceptible to oxidative injury than nuclear DNA (nDNA), although nDNA also undergoes oxidative damage. Environmental exposures and cellular processes can therefore influence mitochondrial genome maintenance, resulting in changes in mtDNA copy number (mtDNAcn) over time. Previous studies have reported associations between peripheral blood mtDNAcn and aging-related outcomes, including age-dependent changes and all-cause mortality, although the direction and magnitude of these associations are not fully consistent across studies [[3], [4], [5]]. Blood-derived mtDNAcn has also been linked to gene expression patterns across multiple organs in humans [6], and cord blood mtDNAcn has been associated with birth weight and other perinatal outcomes [7]. Together, these observations suggest that mtDNAcn represents a dynamic, redox-responsive mitochondrial phenotype whose biological interpretation depends on tissue context and physiological state, rather than a simple or unidirectional biomarker of metabolic health.

Ionizing radiation is a potent inducer of oxidative and redox stress, and radiation-induced perturbation of the mitochondrial genome can alter mtDNA maintenance. Following mtDNA damage, compensatory replication may be observed under certain conditions, while mitochondria retain active base excision repair (BER) pathways for oxidative lesions [8]. Accordingly, mtDNAcn can shift in response to radiation-associated mitochondrial stress through multiple, non-mutually exclusive mechanisms. Radiation-induced changes in mtDNAcn have been reported in cultured cells, animal models, and human populations, and the magnitude and direction of these responses vary across tissues, reflecting differences in mitochondrial content, turnover, and metabolic demand [9]. Such tissue-specific alterations may have long-term physiological consequences, particularly in organs with high mitochondrial energy dependence.

We recently demonstrated that maternal preconception irradiation reduces mtDNAcn in offspring blood, providing evidence of intergenerational disruption of the mitochondrial genome [10]. However, it remains unknown whether paternal irradiation produces similar effects, and whether such intergenerational responses exhibit organ specificity among mitochondria-dependent tissues. In nonhuman biota, intergenerational effects of radiation exposure have been repeatedly reported [11,12]; however, these findings have largely been derived from genomic or epigenomic investigations focusing on the nuclear genome. Although the existence of such effects in humans remains uncertain, hereditary risks of radiation exposure have long been regarded as a major social and ethical concern [13]. The International Commission on Radiological Protection (ICRP) has recommended radiation protection standards based on the assumption that dose to the gonads is proportionally associated with the probability of hereditary effects [14]. Nevertheless, improving the current radiation protection system requires additional data, including evidence regarding radiation-induced impacts on the mitochondrial genome.

In this study, we analyzed mtDNAcn in the brain, heart, and liver of offspring derived from irradiated fathers, irradiated mothers, or both parents. Determining whether preconception irradiation induces organ-specific mtDNAcn dysregulation across generations may provide novel insights at the interface of environmental health, mitochondrial genomics, and redox-mediated developmental regulation.

## 2 Materials and methods

### 2.1 Study design

**Fig. 1:**
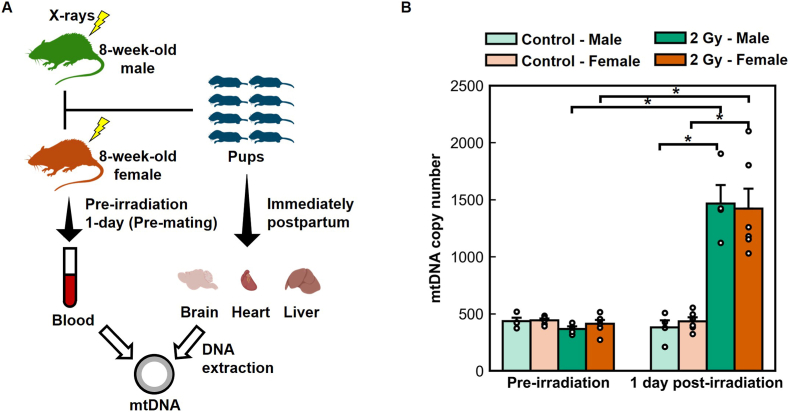
Acute mitochondrial DNA responses *in parents following preconception irradiation.* (A) Experimental design. Eight-week-old male and female C57BL/6 N mice were exposed to 2 Gy of whole-body X-ray irradiation, and peripheral blood was collected immediately before exposure and 1 day after exposure (pre-mating). Newborn pups were sampled at birth for mtDNA analysis in the brain, heart, and liver. Created with BioRender.com. **(B)** Peripheral blood-derived mtDNA copy number in parents. Both sexes exhibited an acute increase in mtDNA copy number 1 day after irradiation, indicating a rapid mitochondrial response to ionizing radiation–induced redox stress. In this panel, mtDNA copy number is expressed as the relative mtDNA:nDNA ratio (dimensionless).

As shown in Fig. 1A, eight-week-old C57BL/6 N male and female mice were exposed to 2 Gy of whole-body X-ray irradiation and assigned to four parental groups: control (sham-irradiated), paternal-only, maternal-only, and dual-irradiation. Peripheral blood was collected from each parent immediately before irradiation and 1 day post-irradiation to quantify mtDNAcn. Newborn pups were euthanized on the day of birth, and the brain, heart, and liver were dissected for mtDNAcn measurement by quantitative PCR (qPCR).

### 2.2 Animals

C57BL/6 N mice were purchased from CLEA Japan (Tokyo, Japan) and maintained under a 12-h light/dark cycle with ad libitum access to food and water. All procedures complied with the ARRIVE guidelines. Animal experiments were approved by the Institutional Animal Care and Use Committee of Hokkaido University and conducted in accordance with the Hokkaido University Regulations for Animal Experimentation (approval no. 24–0008).

### 2.3 Irradiation settings

Male and female mice (8 weeks old) were randomly assigned to the four parental groups, ensuring that each group contained at least two males and at least three females to enable successful breeding. Irradiated mice received a single whole-body dose of 2 Gy of 150 kVp X-rays filtered through 1.0 mm aluminum using an MBR-1520R-4 generator (Hitachi Power Solutions, Ibaraki, Japan) at 1.8 Gy/min. Absorbed dose in air was monitored throughout irradiation using an ionization chamber.

### 2.4 DNA extraction from parental mice

Genomic DNA was extracted from 50 to 100 μL of peripheral blood using the ISOSPIN Blood & Plasma DNA Kit (Nippon Gene, Tokyo, Japan), following the manufacturer's instructions, as described previously [10].

### 2.5 Breeding conditions and litter information

After the second blood collection, each male–female pair was housed for breeding. Females were separated once pregnancy was confirmed and maintained individually until delivery. The mean ± SEM litter sizes were: Control, 7.33 ± 1.33; Paternal-only, 7.33 ± 0.67; Maternal-only, 8.33 ± 0.88; Dual-irradiation, 7.00 ± 1.00. One-way ANOVA showed no significant differences among groups. The mean ± SEM time from pairing to delivery was: Control, 26.67 ± 5.24 days; Paternal-only, 27.67 ± 5.24 days; Maternal-only, 31.00 ± 5.51 days; Dual-irradiation, 29.00 ± 3.06 days (no significant difference).

### 2.6 Weight measurement and tissue collection

Newborn body weight was measured immediately after birth. After euthanasia, the liver, brain, and heart were dissected, blotted to remove blood and fluid, placed in 1.5-mL tubes, and stored at −80 °C. Liver weight was recorded before DNA extraction.

### 2.7 DNA extraction from offspring

Total DNA was extracted from each tissue using the NucleoSpin Tissue Kit (Macherey-Nagel, Düren, Germany) according to the manufacturer's instructions and stored at −20 °C.

### 2.8 Estimation of mtDNAcn

Parental and offspring mtDNAcn were quantified using the Mouse Feeder Cell Quantification Kit (RR290; Takara Bio, Shiga, Japan), which contains primer pairs for mitochondrial and nuclear genes. qPCR was performed on an Applied Biosystems StepOne Real-Time PCR System (Thermo Fisher Scientific, MA, USA), as described previously [10]. The copy number was calculated from the ratio of mitochondrial to nuclear gene amplification.

### 2.9 Statistical analysis

Normality was assessed using the Shapiro–Wilk test. Parental mtDNAcn, newborn body weight, and liver weight were normally distributed and are presented as mean ± SEM and were analyzed using parametric tests. Tissue mtDNAcn values in offspring were non-normally distributed and are shown as box-and-whisker plots (median and interquartile range) and were analyzed using non-parametric tests.

In parental analyses, mtDNAcn at pre-irradiation and 1 day post-irradiation were compared using paired t-tests. For two-group comparisons between control and irradiated parents, or for sex-stratified comparisons where unequal variances were anticipated, Welch's t-tests were applied.

In offspring, comparisons among the four parental groups were conducted using omnibus tests. Differences in tissue mtDNAcn were evaluated using the Kruskal–Wallis test followed by Dunn's post hoc test. Differences in newborn body weight and liver weight were analyzed using one-way ANOVA followed by Tukey–Kramer multiple comparison testing.

Inter-organ correlations in mtDNAcn (brain, heart, and liver) were assessed within each parental group using Spearman's rank correlation coefficients, and correlation coefficients were compared between groups using Fisher's z tests. Pearson's correlation was used to assess the association between body weight and liver weight, whereas Spearman's correlation was used for associations between tissue mtDNAcn and body or liver weight.

Statistical significance was set at p < 0.05. Bonferroni-adjusted thresholds were applied where appropriate to correct for multiple testing. All analyses were performed using Python (Anaconda distribution, version 23.7.4).

## 3 Results

### 3.1 Parental mtDNA responses to preconception irradiation

In parental mice (Fig. 1B), peripheral blood-derived mtDNAcn was significantly higher 1 day after exposure to 2 Gy of single whole-body X-ray irradiation in both males (Welch's *t*-test, *p* = 0.030) and females (*p* = 0.019) compared with sex-matched non-irradiated controls. Within the irradiated groups, mtDNAcn also increased significantly from pre-to post-exposure in males (paired *t*-test, *p* = 0.035) and females (*p* = 0.026). No sex differences were detected at either time point. These observations indicate that preconception irradiation elicits an acute systemic mitochondrial response in both sexes.

### 3.2 Organ-specific and parent-of-origin mtDNA depletion in newborn offspring

**Fig. 2:**
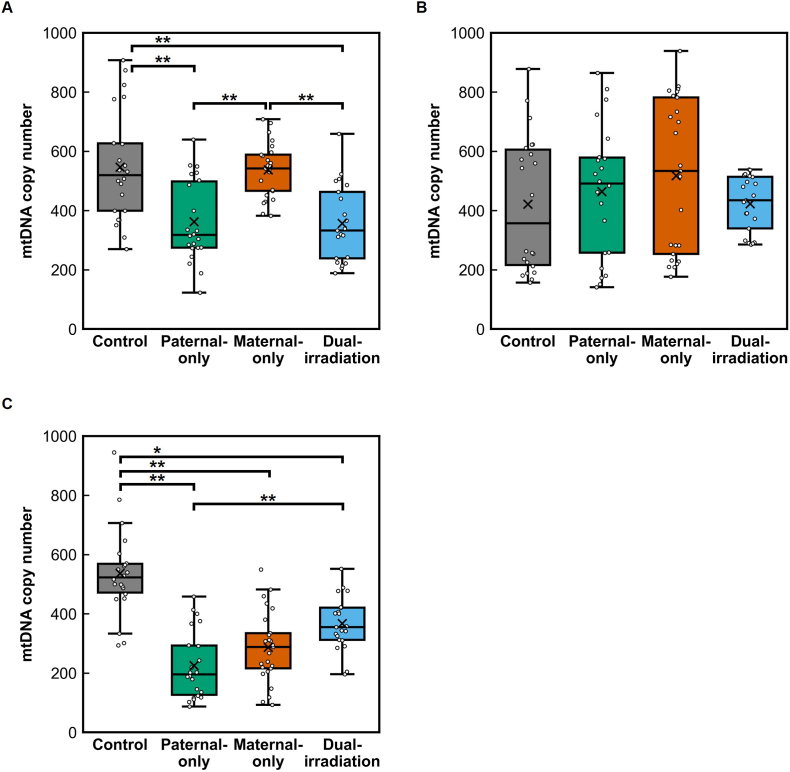
Organ-specific and parent-of-origin mitochondrial DNA depletion in newborn offspring. **(A**–**C)** mtDNA copy number in the brain **(A)**, heart **(B)**, and liver **(C)** across control, paternal-irradiated, maternal-irradiated, and dual-irradiated lineages. Brain mtDNA depletion occurred in paternal- and dual-irradiation lineages, heart mtDNA copy number remained unchanged across all lineages, and liver mtDNA copy number showed the most pronounced depletion in all irradiated lineages. In all panels, mtDNA copy number is expressed as the relative mtDNA:nDNA ratio (dimensionless).

In the offspring (Fig. 2), mtDNAcn exhibited distinct organ-dependent and parent-of-origin-dependent patterns in response to parental irradiation. In the brain, mtDNAcn was significantly reduced in offspring of paternally irradiated parents (*p* = 0.006) and dually irradiated parents (*p* = 0.003) compared with controls (Kruskal–Wallis test followed by Dunn's post hoc test), whereas maternal irradiation alone did not produce a detectable change. Notably, mtDNAcn in the paternal-only and dual-irradiation groups was also significantly lower than in the maternal-only group (both *p* < 0.001). In the heart, no significant differences in mtDNAcn were detected among the four parental groups. In the liver, mtDNAcn was significantly reduced in the paternal-only (*p* < 0.001), maternal-only (*p* < 0.001), and dual-irradiation (*p* = 0.020) groups compared with controls (Kruskal–Wallis with Dunn's test), with the paternal-only group showing a significantly greater reduction than the dual-irradiation group (*p* = 0.007). These findings demonstrate that intergenerational mtDNA depletion is strongly organ-specific, with the liver showing the greatest susceptibility and the brain exhibiting a clear paternal-origin effect.

### 3.3 Lack of inter-organ mitochondrial coordination in newborn offspring

**Fig. 3:**
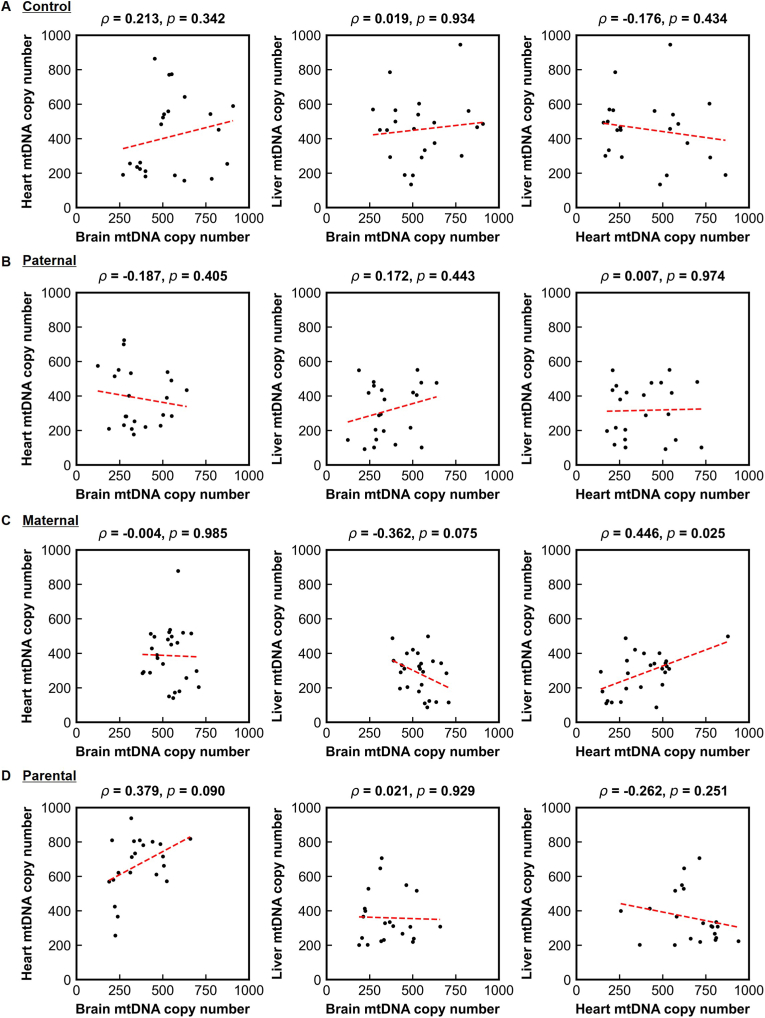
Lack of inter-organ mitochondrial coordination in offspring. **(A**–**D)** Correlation matrices showing relationships in mtDNA copy number among the brain, heart, and liver in control **(A)**, paternal-irradiated **(B)**, maternal-irradiated **(C)**, and dual-irradiated lineages **(D)**. No significant inter-organ correlations were observed in any lineage, indicating that each organ integrates preconception irradiation–induced mitochondrial perturbation independently. As 12 outcome measures were tested against one hypothesized predictor, a Bonferroni-adjusted significance threshold of p < 0.004 (=0.05/12) was applied to account for the increased possibility of type I errors due to multiple testing. In all panels, mtDNA copy number is expressed as the relative mtDNA:nDNA ratio (dimensionless).

To determine whether parental irradiation induces coordinated mitochondrial genomic changes across organs, pairwise correlations in mtDNAcn were examined among the brain, heart, and liver within each parental group (Fig. 3). No significant inter-organ correlations were detected in the control, paternal-only, maternal-only, or dual-irradiation lineages (all Spearman's ρ values nonsignificant). After correction for multiple testing (12 comparisons; Bonferroni-adjusted significance threshold *p* < 0.004), all associations remained nonsignificant. Furthermore, Fisher's z tests showed no significant differences in correlation coefficients between groups, indicating that parental irradiation did not alter the degree of inter-organ mitochondrial coupling. These findings demonstrate that each organ integrates germline-transmitted mitochondrial stress independently, without coordinated mtDNAcn changes across the brain, heart, and liver.

### 3.4 Increased neonatal body and liver mass following parental irradiation

**Fig. 4:**
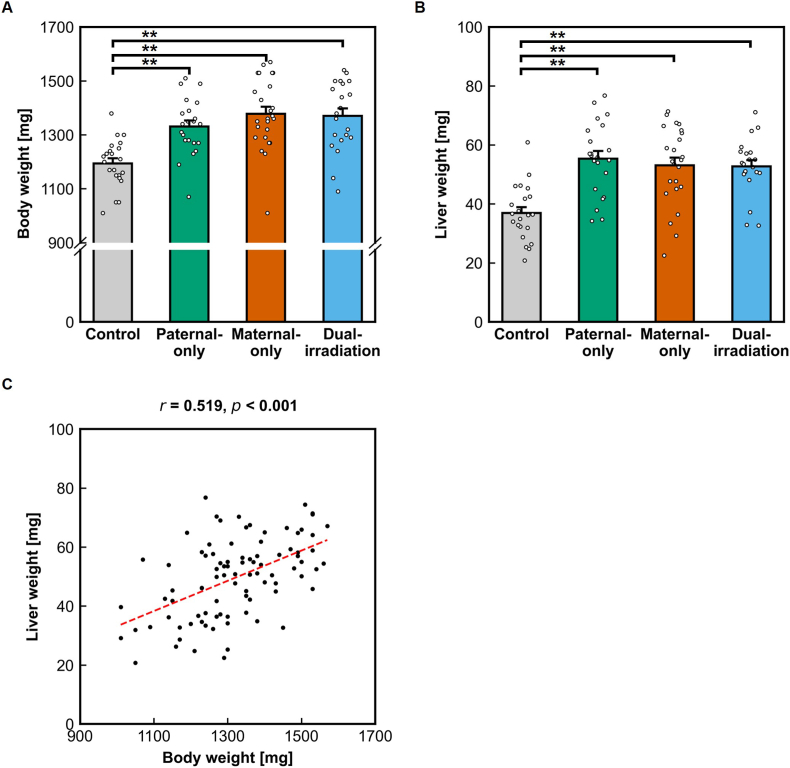
Increased body and liver mass in newborn offspring from irradiated lineages. **(A**–**B)** Newborn body weight **(A)** and liver weight **(B)** were significantly higher in paternal-, maternal-, and dual-irradiated lineages compared with controls, indicating consistent increases in neonatal mass across all irradiated lineages. **(C)** Scatter plot showing a significant positive correlation between body weight and liver weight, demonstrating coordinated neonatal growth-related changes associated with preconception irradiation.

Body weight and liver weight immediately after birth were significantly higher in irradiated offspring than in controls (all *p* < 0.001, one-way ANOVA with Tukey–Kramer test) (Fig. 4A and B). A significant positive correlation was observed between body weight and liver weight (*r* = 0.519, *p* < 0.001), indicating coordinated neonatal growth-related changes in response to preconception irradiation (Fig. 4C).

### 3.5 Associations between mtDNAcn and neonatal body or liver weight

**Fig. 5:**
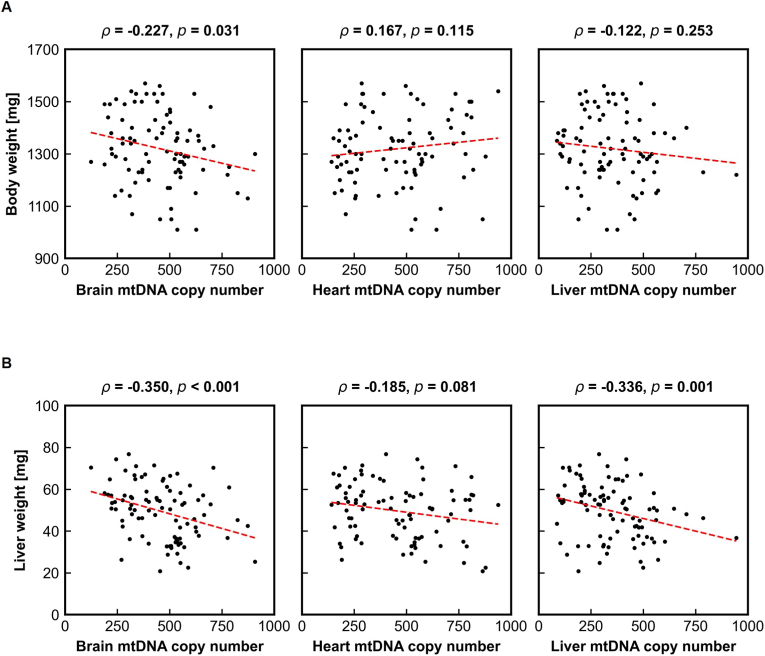
Associations between mtDNA copy number and neonatal liver enlargement. **(A)** Scatter plots showing correlations between neonatal body weight and mtDNA copy number in the brain, heart, and liver. No significant associations were observed, indicating that body weight is not directly linked to organ-specific mtDNA copy number. **(B)** Scatter plots showing correlations between neonatal liver weight and mtDNA copy number in the brain, heart, and liver. Significant negative correlations were detected for brain and liver mtDNA copy number, demonstrating that reduced mtDNA copy number in these organs is associated with increased neonatal liver weight. As 6 outcome measures were tested against one hypothesized predictor, a Bonferroni-adjusted significance threshold of p < 0.008 (=0.05/6) was applied. In all panels, mtDNA copy number is expressed as the relative mtDNA:nDNA ratio (dimensionless).

Fig. 5 shows the relationships between organ-specific mtDNAcn and neonatal body or liver weight, as evaluated using Spearman's rank correlation. Body weight showed no significant associations with mtDNAcn in the brain (*ρ* = −0.227, *p* = 0.031), heart (*ρ* = 0.167, *p* = 0.115), or liver (*ρ* = −0.122, *p* = 0.253); importantly, none of these associations reached significance under the Bonferroni-adjusted threshold, indicating that whole-body growth is not directly linked to mitochondrial genomic status in individual organs.

In contrast, liver weight exhibited distinct organ-specific correlations. Liver weight was weakly but significantly negatively correlated with brain mtDNAcn (*ρ* = −0.350, *p* < 0.001) and with liver mtDNAcn (*ρ* = −0.336, *p* = 0.001), whereas no association was observed with heart mtDNAcn (*ρ* = −0.185, *p* = 0.081). These findings indicate that alterations in mtDNAcn—particularly in the brain and liver—are associated with increased neonatal liver weight, whereas cardiac mtDNAcn remains unrelated to early-life liver enlargement.

### 3.6 Multiple regression analysis of predictors of neonatal liver weight

To identify independent predictors of neonatal liver weight, we constructed a multiple linear regression model using seven explanatory variables: paternal pre-exposure mtDNAcn, maternal pre-exposure mtDNAcn, paternal irradiation dose, maternal irradiation dose, offspring brain mtDNAcn, offspring heart mtDNAcn, and offspring liver mtDNAcn (Table 1). The overall model was statistically significant (*p* < 0.001) and explained 39.7% of the variance in liver weight (R^2^ = 0.397, adjusted R^2^ = 0.345).

**Table 1:** Multiple linear regression analysis identifying independent predictors of neonatal liver weight.

Parameter	Coefficient	95% CI (Lower)	95% CI (Upper)	p-value
**Intercept**	101.639	60.152	143.127	<0.001
**Father mtDNA copy number (Pre-irradiation)**	−0.014	−0.078	0.050	0.672
**Mother mtDNA copy number (Pre-irradiation)**	−0.066	−0.112	−0.019	0.006
**Father dose**	−0.324	−4.332	3.683	0.873
**Mother dose**	2.136	−0.243	4.516	0.078
**Brain mtDNA copy number**	−0.017	−0.034	0.000	0.051
**Heart mtDNA copy number**	−0.005	−0.018	0.008	0.474
**Liver mtDNA copy number**	−0.029	−0.045	−0.013	0.001

∗mtDNA copy number denotes the relative mtDNA:nDNA ratio (dimensionless).

Among the seven predictors, maternal pre-exposure mtDNAcn and offspring hepatic mtDNAcn were significant independent predictors of liver weight. Higher maternal mtDNAcn prior to irradiation was associated with lower neonatal liver weight, and higher hepatic mtDNAcn in offspring was similarly associated with lower liver weight. Offspring brain mtDNAcn showed a negative trend but did not reach statistical significance. None of the remaining variables, including parental irradiation dose or offspring heart mtDNAcn, contributed significantly to the model.

These results indicate that increased neonatal liver weight is influenced independently by maternal mitochondrial status before irradiation and by offspring liver-specific mitochondrial genomic integrity, highlighting the combined germline and organ-specific components of mitochondrial programming following preconception irradiation.

To assess whether variation in delivery timing acted as a potential covariate, correlations between time-to-delivery and neonatal body weight, liver weight, and tissue mtDNA copy number were examined within each irradiation lineage; the results are shown in Supplementary Fig. S1–S4.

## 4 Discussion

Ionizing radiation is a well-established inducer of redox stress, and mitochondria are among the primary cellular structures responsive to oxidative and other forms of radiation-induced stress [3]. In addition to oxidative base damage, ionizing radiation can induce DNA double-strand breaks (DSBs). Because mtDNA lacks canonical DSB repair pathways, such lesions may have biological consequences distinct from redox-mediated damage and could contribute to the observed alterations in mtDNAcn. In this study, we show that preconception exposure to X-rays is associated with an acute increase in mtDNAcn in parents, consistent with compensatory replication, and with intergenerational and organ-specific alterations in mtDNAcn in their offspring. By integrating parental mitochondrial responses, organ-dependent susceptibility, neonatal growth-related traits, and multivariable modeling, our findings provide novel insights into radiation-induced mitochondrial inheritance.

This study reveals two interconnected aspects of intergenerational responses to parental irradiation. First, mtDNAcn in offspring exhibited clear organ-dependent patterns, with consistent reductions in the liver across all irradiated groups and reductions in the brain only after paternal or dual irradiation, whereas the heart remained unchanged. These findings are consistent with our previous observation that maternal irradiation reduces mtDNAcn in offspring peripheral blood [10] and extend it by demonstrating that such effects manifest differently across organs. The paternal-origin–specific decrease in brain mtDNAcn is particularly notable, as maternal irradiation alone produced no detectable change, indicating a germline parent-of-origin-dependent susceptibility in neural mitochondrial maintenance. The presence of paternal exposure–related alterations in offspring mtDNA echoes classical evidence that low-dose paternal irradiation induces germline-transmitted genetic instability across generations [15,16], raising the possibility that such germline-mediated effects extend beyond the nuclear genome to the mitochondrial genome. Although both brain and liver mtDNAcn were negatively associated with liver weight in bivariate analyses, only hepatic mtDNAcn remained significant in multivariable modeling, suggesting that the liver is a principal organ in which alterations in mtDNAcn are associated with neonatal birth outcomes, particularly liver weight.

Second, the offspring exhibited increased body and liver weight, and these traits were linked to mitochondrial genomic status: liver weight correlated positively with body weight but negatively with hepatic mtDNAcn. These observations indicate that parental irradiation can influence offspring mitochondrial genomic maintenance and, in turn, may secondarily affect systemic growth trajectories, reflected here by increased liver and body mass. Importantly, multiple regression analysis identified maternal pre-exposure mtDNAcn and offspring hepatic mtDNAcn as independent predictors of neonatal liver weight, whereas parental irradiation dose and brain or heart mtDNAcn did not remain significant. This finding suggests that maternal mitochondrial status before irradiation and liver-specific mitochondrial genomic integrity in offspring jointly regulate neonatal liver weight. More broadly, these results reinforce the view that early-life perturbations of the mitochondrial genome shape organ-specific vulnerability across mitochondrial physiology and developmental pathogenesis.

The organ-specific alterations in mtDNAcn observed in this study likely reflect intrinsic differences in mitochondrial turnover, redox metabolism, and developmental timing among tissues. The liver, which showed the most pronounced mtDNAcn depletion, is one of the most metabolically active neonatal organs and possesses exceptionally high mitochondrial density and turnover. Such characteristics may render the liver particularly sensitive to germline-transmitted mitochondrial perturbations, amplifying alterations in mtDNAcn into measurable phenotypic associations, such as increased liver weight. In contrast, the brain exhibited a parent-of-origin-dependent response: paternal—but not maternal—irradiation led to reduced mtDNAcn. Because paternal mitochondria are eliminated after fertilization, this effect is unlikely to arise from direct mitochondrial inheritance but rather from radiation-induced alterations in the paternal germline, including epigenetic remodeling or changes in sperm-derived signaling factors that modulate mitochondrial biogenesis in early development [17]. The heart, which showed no detectable mtDNAcn changes, is known to maintain exceptionally robust mitochondrial quality-control mechanisms and efficient redox buffering owing to its high metabolic demand, features that may protect cardiac mitochondria from germline-derived stress signals [18]. Together, these mechanistic considerations support a model in which germline perturbations interact with organ-specific mitochondrial biology to generate distinct susceptibility landscapes across tissues.

While the paternal effects observed in the present study cannot be explained by direct mitochondrial inheritance, they are consistent with prior experimental evidence indicating that ionizing radiation can induce persistent epigenetic alterations in the male germline [19]. Experimental studies have reported radiation-associated changes in DNA methylation and chromatin-related regulation, as well as antioxidant-mediated rescue effects, supporting the involvement of redox-sensitive epigenetic pathways in the transmission of radiation-induced phenotypes [20]. Although these studies were not conducted using a preconception exposure design, they provide a biologically plausible context in which radiation-induced redox stress may influence paternal transmission via epigenetic mechanisms rather than mitochondrial inheritance. Importantly, the present study does not directly assess epigenetic modifications; therefore, these mechanisms remain speculative and warrant direct investigation in future studies.

Beyond these organ-specific patterns, our multivariable regression analysis provides additional insight into how germline and organ-intrinsic mitochondrial factors jointly shape neonatal growth-related traits (neonatal body weight and liver weight). Maternal pre-exposure mtDNAcn emerged as an independent negative predictor of neonatal liver weight, suggesting that maternal mitochondrial status prior to irradiation may contribute to developmental programming in the offspring. Because maternal mtDNAcn was measured in peripheral blood rather than oocytes, this association should be interpreted as reflecting maternal systemic mitochondrial status rather than direct oocyte mitochondrial content. Direct measurements in oocytes will be required to test whether oocyte mitochondrial parameters mediate the observed relationship. Oocytes contain the overwhelming majority of the mitochondria transmitted to the embryo, and their redox state and mitochondrial integrity influence early cleavage events, mitochondrial segregation, and metabolic set points [21]. Thus, elevated maternal mtDNAcn prior to irradiation may reflect a shift in mitochondrial biogenesis or turnover that is associated with altered liver growth trajectories in the offspring. Offspring hepatic mtDNAcn was also an independent negative predictor of liver weight, indicating that the degree of liver-specific mtDNAcn dysregulation directly influences increased neonatal liver weight. The fact that hepatic mtDNAcn—but not brain or heart mtDNAcn—remained significant in the multivariable model further underscores the liver's central role as the primary organ where mtDNAcn dysregulation is translated into a metabolic phenotype. Together, these findings demonstrate that both maternal mitochondrial status before conception and the offspring's own hepatic mitochondrial integrity independently regulate neonatal liver mass, providing mechanistic support for a germline-to-organ axis of mitochondrial programming following parental irradiation.

Although no hereditary effects of radiation have been demonstrated in humans [[22], [23], [24]], accumulating evidence indicates that mitochondrial phenotypes may capture environmentally induced stress across generations. A recent birth cohort study reported that mtDNA copy number in peripheral and cord blood correlates with perinatal outcomes, highlighting mtDNAcn as a candidate indicator of maternal–offspring metabolic coupling [7]. Unlike nuclear DNA mutations, mtDNA copy number reflects a dynamic and environmentally responsive mitochondrial phenotype, shaped by changes in biogenesis, turnover, and redox state, and is therefore potentially reversible [25]. In this context, radiation-induced alterations in mtDNAcn may represent not a permanent genetic legacy but a modifiable metabolic imprint that influences the developmental resilience of subsequent generations. These experimental findings align with observations from radiation-exposed human populations, including the Hiroshima and Nagasaki cohorts, where no increases in hereditary disease have been detected [26], and together suggest that mitochondrial endpoints could provide sensitive and biologically meaningful markers for future radiological risk assessment.

Several limitations should be considered when interpreting these findings. First, mtDNAcn was used as a quantitative marker of mitochondrial genomic status, but we did not assess mtDNA sequence integrity, mutation burden, deletions, or replication intermediates. These additional layers of mitochondrial genome integrity and regulation could further refine the mechanistic basis of the observed phenotypes. Second, functional assays of mitochondrial activity—such as respiratory chain capacity, ROS generation, or metabolomic profiling—were beyond the scope of this study but would help clarify how mtDNAcn depletion translates into altered metabolic output in specific organs. Third, although our design distinguishes paternal and maternal contributions, the underlying germline mechanisms driving these parent-of-origin effects remain unresolved. We did not assess mtDNAcn in gonadal tissues of parental mice at the acute post-irradiation time point. Direct evaluation of testicular and ovarian mtDNA dynamics will be necessary to clarify how preconception irradiation perturbs germline mitochondrial status and contributes to the observed intergenerational effects. Integrating germline epigenomics and early embryonic mitochondrial dynamics will be needed to identify the pathways through which preconception irradiation shapes organ-specific mitochondrial phenotypes in offspring. Finally, while our study focused on birth outcomes, longitudinal analyses will be required to determine whether these mitochondrial alterations persist beyond the neonatal period or contribute to later-life susceptibility to metabolic disease.

**Fig. 6:**
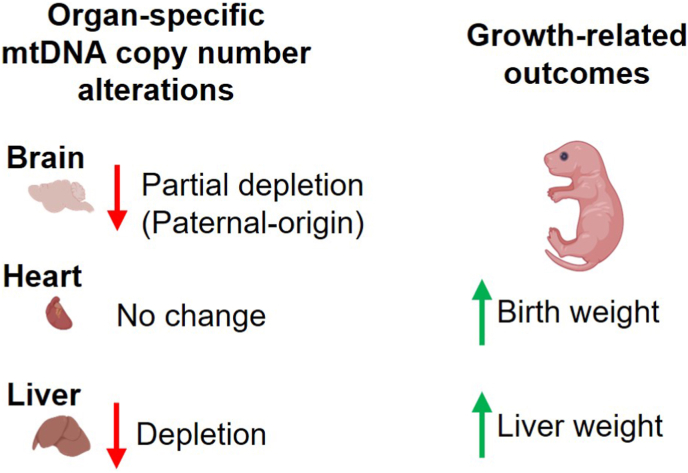
Neonatal organ-specific mtDNA copy number alterations following parental preconception irradiation and growth-related traits. Preconception irradiation acts as a redox stressor on parental germ cells, leading to offspring with brain mtDNA depletion in paternal- and dual-irradiated lineages and liver mtDNA depletion in all irradiated lineages. These mtDNA copy number alterations are associated with increased body weight and liver weight at birth, highlighting a possible link between intergenerational mtDNA copy number dysregulation and neonatal growth-related traits. Created with BioRender.com.

In conclusion, preconception irradiation perturbs mitochondrial genomic regulation at multiple biological levels—initiating acute mitochondrial responses in parents, establishing organ-specific and parent-of-origin-dependent alterations in mtDNAcn in offspring, and shaping neonatal phenotypes (increased liver and body weight). The integrative model presented in Fig. 6 summarizes this germline-to-organ axis of mitochondrial programming, in which radiation-induced perturbations in parental mitochondrial status influence tissue-specific mtDNA maintenance and neonatal growth-related traits. By revealing that maternal pre-exposure mtDNAcn and offspring hepatic mtDNAcn independently predict liver weight, our study highlights mtDNAcn dysregulation as a biologically meaningful yet previously underrecognized contributor to intergenerational responses to radiation. More broadly, these results underscore the importance of incorporating mitochondrial endpoints into evaluations of redox-mediated stress, developmental programming, and hereditary health risks, and suggest that mitochondrial genome dynamics should be considered in future refinements of radiation protection frameworks.

## Funding

This work was supported by JST 10.13039/501100020964FOREST Program, Grant Number JPMJFR211E.

## CRediT authorship contribution statement

**Ryosuke Seino:** Data curation, Formal analysis, Investigation, Validation, Writing – original draft. **Hisanori Fukunaga:** Conceptualization, Data curation, Formal analysis, Funding acquisition, Methodology, Supervision, Writing – original draft, Writing – review & editing.

## Declaration of competing interest

The authors declare no competing interests.

## Data Availability

Data will be made available on request.
