# Supplementary material for: Intergenerational and organ-specific alterations in mitochondrial DNA copy number following preconception irradiation
Source: Redox Biol. 2026 Jan 30;90:104054. doi: 10.1016/j.redox.2026.104054 (PMC12906075; doi:10.1016/j.redox.2026.104054)
Supplement: Multimedia component 1 [file mmc1.pdf]

Supplementary Data for

Intergenerational and organ-specific alterations in mitochondrial DNA copy number following preconception irradiation

Ryosuke Seino[1] and Hisanori Fukunaga[1,2,*]

[1] Department of Biomedical Science and Engineering, Faculty of Health Sciences, Hokkaido University, Sapporo, Japan

[2] Center for Environmental and Health Sciences, Hokkaido University, Sapporo, Hokkaido, Japan

* Email: hisanori.fukunaga@hs.hokudai.ac.jp

This file includes:

Supplementary Tables S1–6

Supplementary Figures S1–4

Supplementary Table S1. Changes in paternal mitochondrial DNA copy number before and after irradiation.

Dose	ID	mtDNA copy number	
		Pre-irradiation	1 day post-irradiation
**Control**	**Control male 1**	425	377
	**Control male 2**	422	427
	**Control male 3**	375	210
	**Control male 4**	519	508
**2 Gy**	**2 Gy male 1**	315	1412
	**2 Gy male 2**	422	1424
	**2 Gy male 3**	343	1121
	**2 Gy male 4**	385	1904

*mtDNA copy number denotes the relative mtDNA:nDNA ratio (dimensionless).

Supplementary Table S2. Changes in maternal mitochondrial DNA copy number before and after irradiation.

Dose	ID	mtDNA copy number	
		Pre-irradiation	1 day post-irradiation
**Control**	**Control female 1**	410	382
	**Control female 2**	440	398
	**Control female 3**	484	495
	**Control female 4**	391	554
	**Control female 5**	469	323
	**Control female 6**	463	456
**2 Gy**	**2 Gy female 1**	443	1181
	**2 Gy female 2**	379	1259
	**2 Gy female 3**	515	1030
	**2 Gy female 4**	455	1153
	**2 Gy female 5**	271	1803
	**2 Gy female 6**	412	2101

*mtDNA copy number denotes the relative mtDNA:nDNA ratio (dimensionless).

Supplementary Table S3. Birth outcomes and mtDNA copy number in pups obtained from the control group.

Group	Pup ID	Brain mtDNA copy number	Heart mtDNA copy number	Liver mtDNA copy number	Weight [mg]	Liver weight [mg]
**Control 1**	**Pup 1**	824	452	561	1150	45.3
	**Pup 2**	536	771	603	1380	34.9
	**Pup 3**	873	254	467	1130	42.5
	**Pup 4**	908	590	487	1300	25.3
	**Pup 5**	776	543	945	1220	36.7
	**Pup 6**	531	559	540	1300	36.4
**Control 2**	**Pup 1**	784	167	301	1250	60.9
	**Pup 2**	309	257	451	1140	36.2
	**Pup 3**	399	181	500	1200	34.0
	**Pup 4**	368	225	785	1230	46.2
	**Pup 5**	369	262	293	1240	37.6
	**Pup 6**	269	191	570	1160	26.3
	**Pup 7**	624	157	493	1150	41.8
	**Pup 8**	400	213	565	1270	49.9
	**Pup 9**	569	188	333	1260	32.3
	**Pup 10**	351	236	450	1230	46.1
**Control 3**	**Pup 1**	491	484	135	1170	28.7
	**Pup 2**	627	643	375	1010	39.7
	**Pup 3**	552	774	291	1210	24.8
	**Pup 4**	454	864	190	1050	20.8
	**Pup 5**	507	542	458	1050	31.9
	**Pup 6**	500	523	188	1170	32.8

*mtDNA copy number denotes the relative mtDNA:nDNA ratio (dimensionless).

Supplementary Table S4. Birth outcomes and mtDNA copy number in pups obtained from the paternal-only irradiated group.

Group	Pup ID	Brain mtDNA copy number	Heart mtDNA copy number	Liver mtDNA copy number	Weight [mg]	Liver weight [mg]
**Paternal-only 1**	**Pup 1**	275	724	102	1340	54.8
	**Pup 2**	123	575	145	1270	70.4
	**Pup 3**	304	402	288	1240	76.8
	**Pup 4**	315	533	294	1340	56.4
	**Pup 5**	320	209	434	1070	55.8
	**Pup 6**	335	253	380	1190	64.9
	**Pup 7**	487	228	216	1310	61.2
	**Pup 8**	331	177	198	1350	66.7
**Paternal-only 2**	**Pup 1**	275	231	460	1360	42.2
	**Pup 2**	188	209	549	1490	58.2
	**Pup 3**	244	552	418	1510	74.4
	**Pup 4**	273	700	482	1430	45.0
	**Pup 5**	288	282	148	1280	69.0
	**Pup 6**	221	515	93	1380	57.1
	**Pup 7**	284	282	205	1490	56.9
	**Pup 8**	400	220	118	1390	54.0
**Paternal-only 3**	**Pup 1**	552	284	102	1300	34.2
	**Pup 2**	528	539	552	1230	34.7
	**Pup 3**	639	435	477	1350	37.8
	**Pup 4**	501	290	420	1420	50.5
	**Pup 5**	549	490	478	1270	41.7
	**Pup 6**	524	390	406	1280	54.6

*mtDNA copy number denotes the relative mtDNA:nDNA ratio (dimensionless).

Supplementary Table S5. Birth outcomes and mtDNA copy number in pups obtained from the maternal-only irradiated group.

Group	Pup ID	Brain mtDNA copy number	Heart mtDNA copy number	Liver mtDNA copy number	Weight [mg]	Liver weight [mg]
**Maternal-only 1**	**Pup 1**	437	429	332	1360	55.9
	**Pup 2**	466	391	401	1530	64.1
	**Pup 3**	451	496	312	1560	54.4
	**Pup 4**	430	513	291	1430	47.7
	**Pup 5**	382	285	488	1570	67.1
	**Pup 6**	389	292	358	1530	58.9
	**Pup 7**	426	288	196	1530	71.4
**Maternal-only 2**	**Pup 1**	501	339	422	1460	66.5
	**Pup 2**	470	373	205	1400	65.0
	**Pup 3**	696	298	285	1480	52.8
	**Pup 4**	596	180	124	1390	61.8
	**Pup 5**	636	258	118	1370	54.9
	**Pup 6**	552	498	219	1230	58.3
	**Pup 7**	536	151	179	1360	67.5
	**Pup 8**	708	205	115	1330	70.3
**Maternal-only 3**	**Pup 1**	584	462	87	1350	43.5
	**Pup 2**	560	141	293	1270	52.6
	**Pup 3**	570	173	111	1290	53.5
	**Pup 4**	530	481	401	1350	45.1
	**Pup 5**	617	520	355	1530	45.8
	**Pup 6**	549	451	341	1270	36.4
	**Pup 7**	665	516	343	1320	47.7
	**Pup 8**	537	523	325	1010	29.2
	**Pup 9**	542	537	310	1240	33.4
	**Pup 10**	588	878	499	1290	22.5

*mtDNA copy number denotes the relative mtDNA:nDNA ratio (dimensionless).

Supplementary Table S6. Birth outcomes and mtDNA copy number in pups obtained from the parental irradiated group.

Group	Pup ID	Brain mtDNA copy number	Heart mtDNA copy number	Liver mtDNA copy number	Weight [mg]	Liver weight [mg]
**Dual-irradiation 1**	**Pup 1**	523	572	517	1280	37.2
	**Pup 2**	311	623	647	1360	50.7
	**Pup 3**	319	712	706	1400	48.1
	**Pup 4**	242	622	529	1290	50.5
	**Pup 5**	463	611	550	1380	51.1
	**Pup 6**	501	716	219	1470	59.3
	**Pup 7**	659	819	308	1240	57.1
	**Pup 8**	506	662	239	1090	32.9
	**Pup 9**	387	782	312	1300	55.0
**Dual-irradiation 2**	**Pup 1**	316	939	224	1540	52.5
	**Pup 2**	366	810	335	1500	65.9
	**Pup 3**	339	734	328	1530	71.1
	**Pup 4**	441	801	267	1500	55.0
	**Pup 5**	486	788	308	1450	32.7
	**Pup 6**	332	805	231	1500	50.1
**Dual-irradiation 3**	**Pup 1**	189	569	202	1260	57.7
	**Pup 2**	238	366	203	1140	53.9
	**Pup 3**	221	424	414	1320	50.8
	**Pup 4**	205	810	243	1440	57.4
	**Pup 5**	225	257	400	1300	53.5
	**Pup 6**	214	580	367	1490	64.8

*mtDNA copy number denotes the relative mtDNA:nDNA ratio (dimensionless).

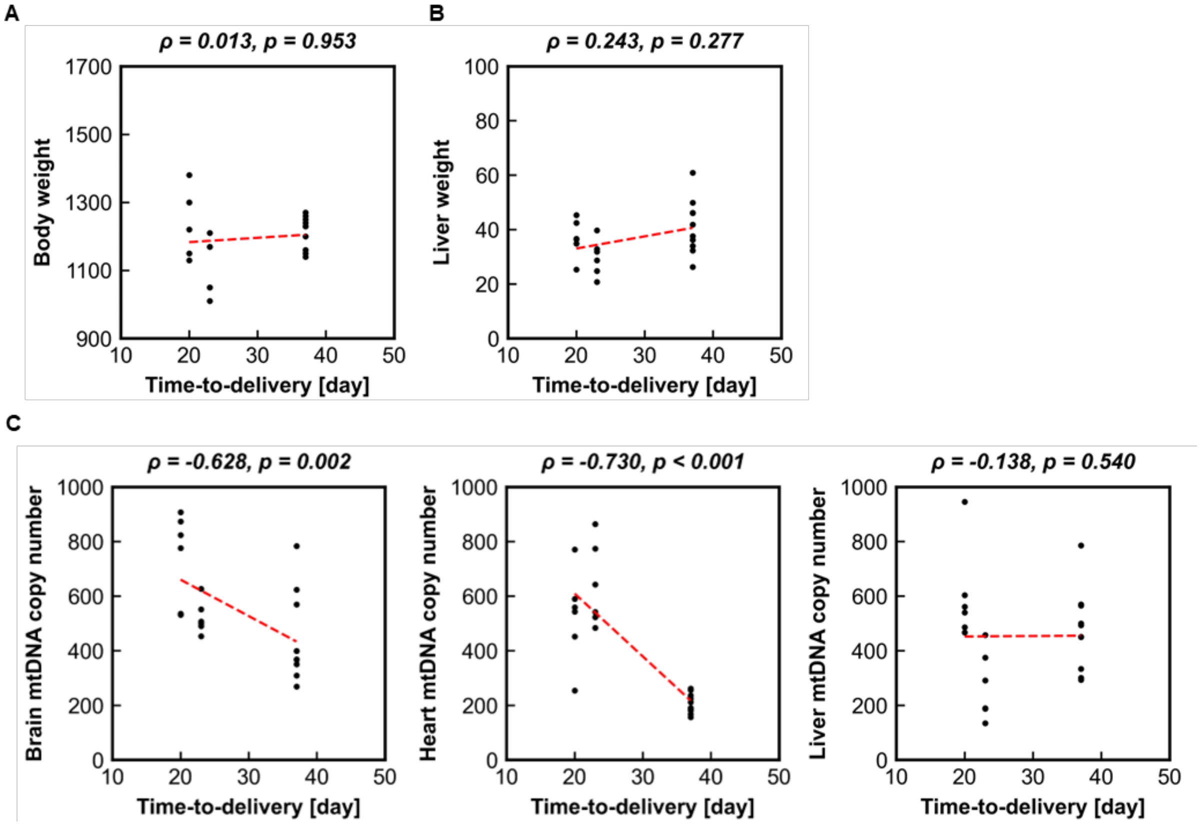

Supplementary Figure S1. Correlations between time-to-delivery and neonatal parameters in the control lineage.

Correlation analyses between time-to-delivery and neonatal body weight, liver weight, and tissue mtDNA copy number were performed in the control lineage using Spearman's rank correlation. While significant associations were observed for brain and heart mtDNA copy number, no consistent relationships were detected across growth-related traits or tissues. These findings indicate that delivery timing does not uniformly influence neonatal phenotypes under non-irradiated conditions. In all panels, mtDNA copy number is expressed as the relative mtDNA:nDNA ratio (dimensionless).

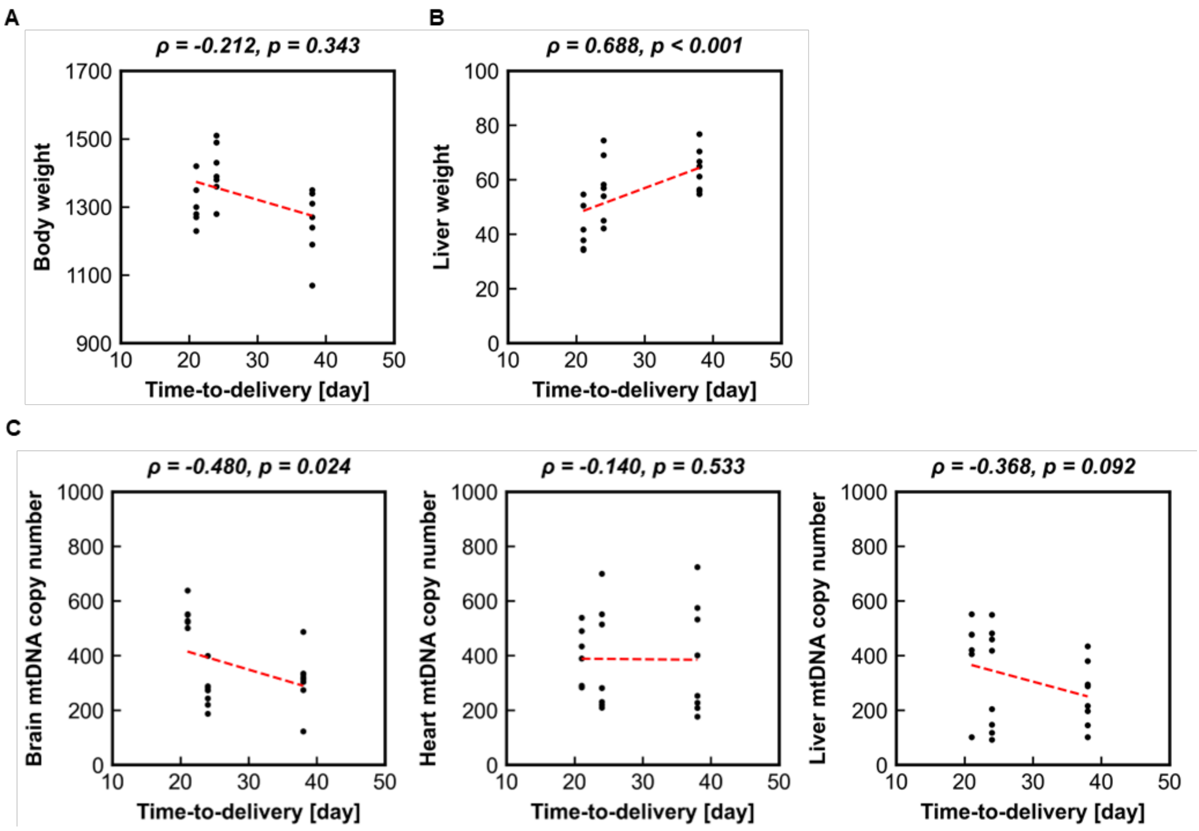

Supplementary Figure S2. Correlations between time-to-delivery and neonatal parameters in the paternal-only irradiation lineage.

Correlation analyses between time-to-delivery and neonatal body weight, liver weight, and tissue mtDNA copy number were performed in the paternal-only irradiation lineage. Although a significant positive association was observed between time-to-delivery and liver weight, correlations with mtDNA copy number were variable and did not show a consistent directional pattern across tissues. These results suggest lineage-specific associations rather than a general covariate effect of delivery timing. In all panels, mtDNA copy number is expressed as the relative mtDNA:nDNA ratio (dimensionless).

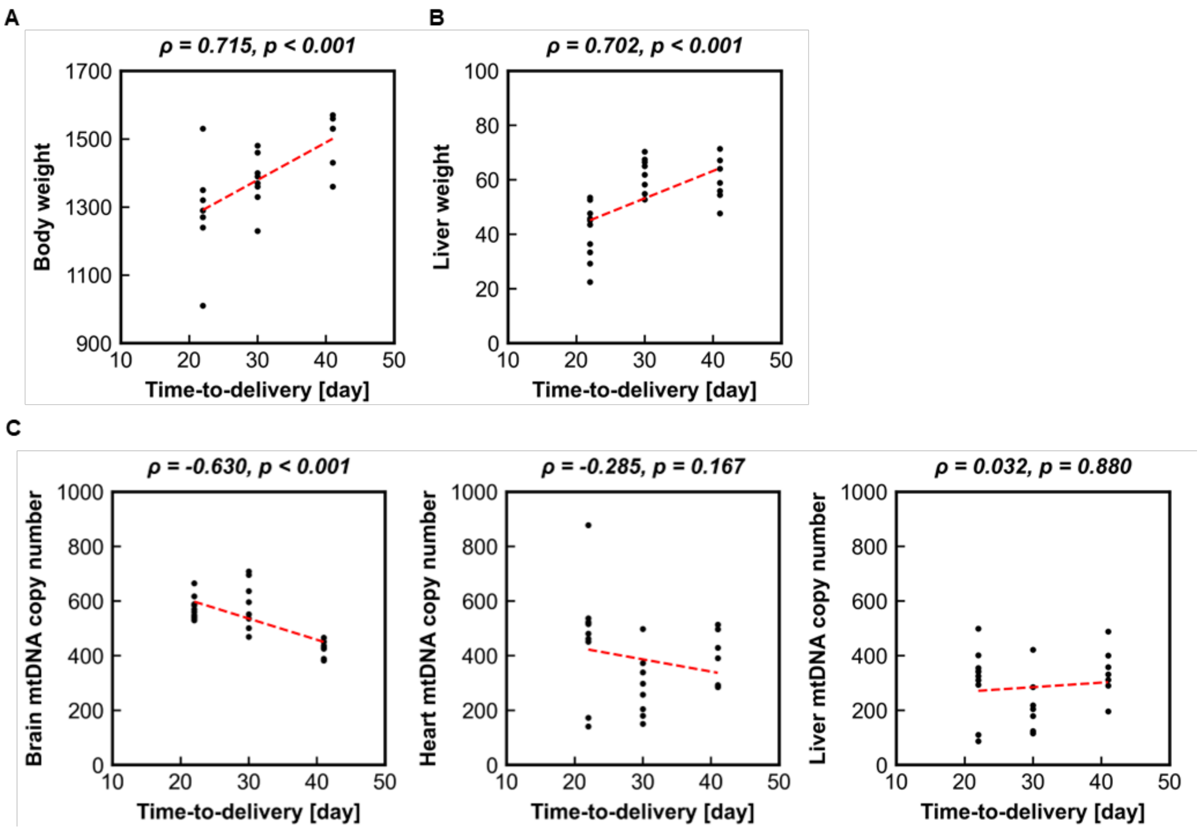

Supplementary Figure S3. Correlations between time-to-delivery and neonatal parameters in the maternal-only irradiation lineage.

Correlation analyses between time-to-delivery and neonatal body weight, liver weight, and tissue mtDNA copy number were conducted in the maternal-only irradiation lineage. Significant positive correlations were observed for body weight and liver weight, whereas associations with tissue mtDNA copy number differed by organ. The presence of both positive and negative associations across outcomes indicates that delivery timing does not act as a uniform determinant of neonatal mitochondrial or growth-related phenotypes. In all panels, mtDNA copy number is expressed as the relative mtDNA:nDNA ratio (dimensionless).

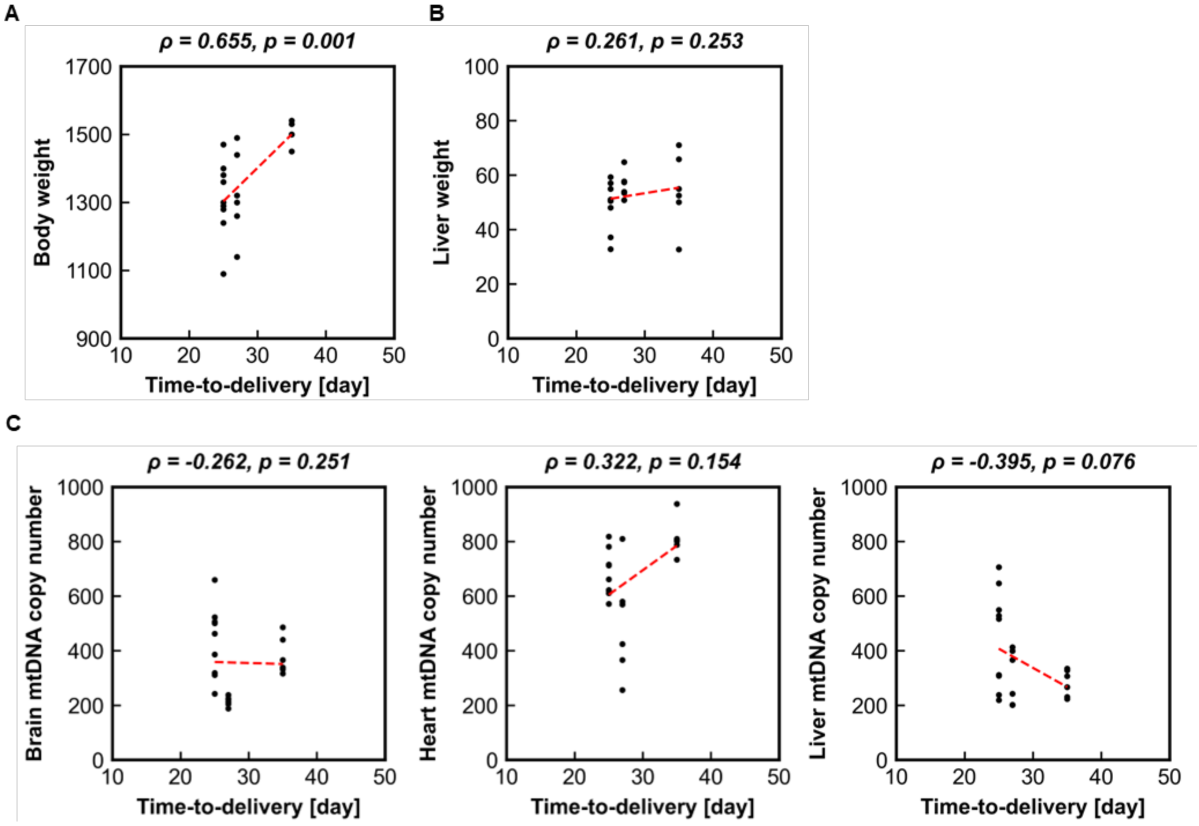

Supplementary Figure S4. Correlations between time-to-delivery and neonatal parameters in the dual-irradiation lineage.

Correlation analyses between time-to-delivery and neonatal body weight, liver weight, and tissue mtDNA copy number were performed in the dual-irradiation lineage. No statistically significant associations were detected across the examined parameters. Together with the lineage-specific patterns observed in other groups, these results indicate that variation in delivery timing does not represent a consistent covariate across irradiation conditions. In all panels, mtDNA copy number is expressed as the relative mtDNA:nDNA ratio (dimensionless).
